# PolDrugs 2025: results of the third edition of the nationwide study on psychoactive substance use in the context of psychiatry and harm reduction

**DOI:** 10.3389/fpsyt.2025.1591658

**Published:** 2025-06-27

**Authors:** Julia Marek, Magdalena Domek-Gumprecht, Agata Macionga, Sandra Szafoni, Gniewko Więckiewicz

**Affiliations:** ^1^ Students’ Scientific Circle, Department of Psychiatry, Faculty of Medical Sciences in Zabrze, Medical University of Silesia, Tarnowskie Góry, Poland; ^2^ Department of Psychiatry, Faculty of Medical Sciences in Zabrze, Medical University of Silesia, Tarnowskie Góry, Poland

**Keywords:** drugs, PolDrugs, psychoactive substances, psilocybin, DMT, marijuana, MDMA, psychiatry

## Abstract

**Introduction:**

PolDrugs is a biennial epidemiological study aimed at analyzing patterns of mostly illicit psychoactive substance use in Poland in the context of psychiatry and harm reduction. This survey was held for the third time, and its results were compared to the last two editions.

**Materials and methods:**

The survey was conducted as an online survey with 37 closed-ended single-choice questions and 3 multiple-choice questions. Respondents were recruited through outreach on social media platforms, primarily Facebook and Instagram, in drug-related groups. Recruitment efforts were supported by activists and advocacy groups who promoted the questionnaire through their own social media networks. The sample consisted of 2,447 people between the ages of 13 and 63 years. Statistical analysis included descriptive statistics only.

**Results:**

The study population (mean age 27 years) was predominantly male and urban. Marijuana was the most common substance used after alcohol, caffeine, and nicotine, though overall consumption was infrequent (35.6% reporting use once every few months or less) and mainly occurred in social settings (50.2%) or at home (52.3%). Notably, 83.6% never tested substance composition, and 51.4% relied on visual estimation for dosing. Sixty percent had neglected daily responsibilities, while 16.8% faced legal issues. Although 70.7% had not sought medical help, nearly half had seen a psychiatrist (primarily for depression), with 41.1% of these having attempted suicide and 70.5% using illicit substances before their initial consultation. Only 40% consistently disclosed their substance use to a physician.

**Conclusions:**

Stimulant use and subsequent medical consultations—particularly for mephedrone derivatives—are rising warranting further investigation. The proportion of respondents who use psychoactive substances alone is increasing, now exceeding 25%. Psychedelic use is declining possibly due to reduced mainstream media attention. The observation also shows a growing acceptance of psychiatric care in Polish society.

## Introduction

PolDrugs is a cyclical research project that aims to provide up-to-date, naturalistic epidemiological and demographic data on recreational users of psychoactive substances in Poland. The project was launched in 2020 and aims to reach the widest possible range of psychoactive substance users. Its main idea is to draw the attention of psychiatrists to the fact that, in addition to patients of addiction treatment clinics, there is also a significant group of psychoactive substance users who are not under the care of these institutions or who come to the doctor for other reasons. Previous editions of the survey were conducted in November and December of 2020 and 2022 and published in 2021 and 2023 (1, 2), and are named after the year of publication. PolDrugs’ results each time have received a lot of attention not only in the psychiatric community (the results of the last edition were presented, among other things, in a full-fledged lecture at the World Psychiatric Association’s World Congress of Psychiatry in September 2023 in Vienna) but also in the media across the country. The widespread interest was due, among other things, to the fact that Poland is a country with a relatively conservative approach to the law on possession of psychoactive substances, which is unique in the European Union and underscores the need for reliable research in this area.

The time that has passed since the last editions of PolDrugs (published in 2023 and 2021) has been characterized by changes that can affect the popularity and use of substances outside the controlled market. This includes cultural, legal, medical and research aspects. The first edition of this manuscript was launched during the COVID-19 pandemic, a time marked by isolation, uncertainty, and heightened vulnerability to substance use. As life gradually returns to a sense of normalcy, it remains crucial to continually assess the evolving landscape of drug use. Today, new challenges, such as economic instability and ongoing global conflicts, pose additional stressors that may drive individuals toward substance misuse. These shifting pressures underscore the persistent and complex nature of drug-related issues in society. In 2024, as a result of traditional media, the issue of recreational use of fentanyl was a particularly frequent topic (3). On 17 April 2023, the Polish Ministry of Health issued a decree amending the list of psychotropic substances, narcotics, and new psychoactive substances aimed at bringing new psychoactive substances appearing on the market under control (4). There has been an increase in opioid poisoning cases in the United States. The challenges of the opioid crisis in the United States of America are influencing the Polish sociopolitical discourse. There has also been an increase in the intensity of public debates over the decriminalization of marijuana inspired by the legalization of the substance in Germany and legislative plans in the Czech Republic. The Parliamentary Team for the Depenalization of Marijuana, formed on 25 September 2024, began work on revising marijuana laws (5). In June of the same year, the U.S. Food and Drug Administration (FDA) rejected an application to allow the use of 3,4-methylenedioxymethamphetamine (MDMA) for the treatment of PTSD (6). In contrast, in December 2024, the Warsaw Medical University received funding for a clinical trial to evaluate the efficacy and safety of a treatment method for drug-resistant depression using psilocybin. Esketamine is a drug available in Poland for patients with drug-resistant depression, and research is also underway into its use in the treatment of bipolar affective disorder (7, 8). At the same time, invariably, doctors of various specialties around the world have had to grapple with the challenge of caring for public health assumptions and the consequences of widespread access to new psychoactive substances (9, 10). In November 2024, the Supreme Audit Office published a report according to which an increase in the number of poisonings with OTC drugs containing psychoactive substances continues in Poland (11). On the other hand, a decrease in the amount of alcoholic beverages consumed per capita in Poland was observed (12). For these reasons, it is necessary to regularly update epidemiological and demographic data. The aim of the study was to assess possible changes and recent trends in the problem of psychoactive substance use in the context of previous PolDrugs editions.

## Materials and methods

### Survey

The online survey included 37 closed-ended single-choice questions and 3 multiple-choice questions. Information was collected on basic demographics, characteristics of contact with psychoactive substances, history of psychiatric treatment, and respondents’ attitudes toward the form of conducting the survey via the Internet. The survey was conducted through Google Forms, a frequently used platform due to its user-friendly interface. With its help in scientific practice, authors can collect and analyze data via the Internet. Before starting the survey, users had to confirm that they voluntarily consented to the release and further processing of the data and, in the case of minors, that they had the consent of their legal guardian. To ensure a sense of security, the survey was completely anonymous. The authors collected neither IP addresses nor email addresses. Since the questions were asked on an online platform, it is not possible to attach the original version of the survey as an appendix to the article, so all questions and the original layout of our survey have been included in the tables in the “Results” section without changing the content and should be treated as such. According to the local regulations, the study, as an anonymous, Internet-based questionnaire, did not require ethical committee acceptance. The anonymized data collected was processed in accordance with the data protection regulations of the Republic of Poland and the European Union.

### Data collection

Data were collected from 11 November to 29 December 2024. A total of 2,450 surveys were submitted, of which 2,447 were correctly completed and included in the analysis. Three surveys were excluded due to lack of consent for data use. The data were to be compared with the results of previous editions of PolDrugs, so they were collected in the same way as before, i.e., via social media (Facebook, Instagram). The survey was shared on sites for users of psychoactive substances and groups related to stimulant use, such as sites about music festivals and club culture, and sites for artists, students, and schoolchildren. Stimulant-related hashtags were also used. Activists participated in the distribution of the survey, mainly from NGOs related to drug policy, and also influencers running social media profiles on psychoactive substance use.

### Statistical analysis

The questionnaire method of data collection used in the PolDrugs survey involves random sampling, which burdens the survey sample by risking bias that could affect the survey results due to low external validity, so the analysis presents only descriptive statistics without conducting an inference analysis to avoid potentially erroneous conclusions.

## Results

The youngest person surveyed was 13 years old, and the oldest was 63 years old. The average age of the respondents was 27 years. Most of the surveyed users of psychoactive substances were male (56.1%), heterosexual (78%), and in an informal relationship (50.6%). The majority of the respondents were residents of large cities (56.1%), with a high school education (50.6%), who were full-time employees (47.3%), and declared monthly earnings of more than PLN 5,000 net (46.4%). Detailed demographic data are provided in **Table 1**.

**Table 1:** Demographic details.

Variable		n	%
Age (years)	13–15	2	0.1
	16–17	37	1.5
	18–24	997	40.7
	25–30	801	32.7
	31–40	520	21.3
	41–63	90	3.7
Gender	Male	1,372	56.1
	Female	1,048	42.8
	Other	27	1.1
Sexual orientation	Heterosexual	1,908	78
	Bisexual	371	15.2
	Homosexual	121	4.9
	Other	47	1.9
Relationship status	In an informal relationship (including engagement)	1,240	50.6
	Single/single	857	35.0
	In a formal relationship (marriage)	253	10.3
	Divorced	28	1.1
	In a polyamorous/polygamous relationship	27	1.1
	Widowed	3	0.1
	Other	39	1.6
Place of residence	Large city (more than 200,000 inhabitants)	1,372	56.1
	Medium city (50,000 to 199,999 inhabitants)	449	18.3
	Small city (up to 49,999 inhabitants)	350	14.3
	Village	276	11.3
Education	I have a doctorate or other academic degree	22	0.9
	Master’s degree or equivalent	478	19.3
	Bachelor’s degree or equivalent	535	21.9
	Medium	1,238	50.6
	Middle school	80	3.3
	Basic	94	3.8
Profession	Pupil: working	86	3.5
	Pupil: unemployed	87	3.6
	Student: working	382	15.6
	Student: unemployed	171	7.0
	Full-time employee	1,157	47.3
	Casual worker	79	3.2
	Retired or pensioner	5	0.2
	Own business	389	15.9
	Unemployed	91	3.7
Net monthly earning (1 EUR = approximately 4.1 PLN)	Not applicable	327	13.4
	Up to 1,000 PLN	65	2.7
	1,000–2,000 PLN	94	3.8
	2,000–3,000 PLN	138	5.6
	3,000–4,000 PLN	278	11.4
	4,000–5,000 PLN	409	16.7
	Over 5,000 PLN	1,136	46.4

The use of nicotine is declared by 1,648 respondents (67.3%), most often taking it in the form of e-cigarettes (liquids) (26.2%). Classic cigarettes, on the other hand, are chosen by 583 respondents (23.8%). A total of 1,783 respondents admit to taking alcohol (72.9%). Most drink it two to three times a month (27.4%) and choose beer (48.2%). What is more, 16.2% of the drinking respondents considered getting help for their alcohol use. Detailed results on nicotine and alcohol use can be found in **Table 2**.

**Table 2:** Detailed results on nicotine and alcohol consumption.

Variables		n	%
Are you taking nicotine?	Yes, mostly classic cigarettes	583	23.8
	Yes, mostly tobacco vaporization (e.g., IQOS system)	288	11.8
	Yes, mostly e-cigarette (liquid)	640	26.2
	Yes, others	137	5.6
	No	799	32.7
Do you use alcohol?	Yes	1,783	72.9
	No	664	27.1
How often do you consume alcohol? (n = 1,783)	Once every few months or less frequently	230	12.9
	Once a month	275	15.4
	2–3 times a month	487	27.3
	4–5 times a month	320	17.9
	6–8 times a month	167	9.4
	9–10 times a month	76	4.3
	More than 10 times a month	228	12.8
What type of alcohol do you consume most often? (n = 1,783)	Beer	860	48.2
	Wine	334	18.7
	Vodka or other spirits	254	14.2
	Drinks	245	13.7
	Liqueurs including flavored vodkas (20%–30% alcohol content)	72	4.0
	Other	18	1.0
Have you ever thought about getting help because of your alcohol consumption? (n = 1,783)	Yes	288	16.2
	No	1,495	83.8

The average age of first contact with psychoactive substances is 19 years, while the lowest recorded age of initiation is 4 years. The most commonly used psychoactive substance other than alcohol, caffeine, and nicotine is marijuana, which 97.9% of the respondents in their lifetime have used, and 85% of the respondents have used it in the past 12 months. MDMA comes in second place (similarly, 75.9% and 42.1%). Psychoactive substances are most often used once every few months or less often (35.6%), with friends (50.2%), and in one’s own home (52.3%). Most users buy psychoactive substances from friends (47.2%) and spend up to PLN 100 a month on them (31.1%). It was found that 83.6% of the respondents never test their psychoactive substances with colorimetric reagents or in a laboratory, and 51.4% of the respondents measure the dose by visual observation. A total of 86.5% of the respondents have tried to educate themselves about the safety and risks of psychoactive substances (including scientific articles, YouTube, and Instagram). In total, 33.6% of the respondents never use dietary supplements to support recovery from psychoactive substance use. A total of 60.0% of the respondents have neglected daily chores at least once in their lives due to the use of psychoactive substances. In total, 16.8% of the respondents had problems with the law due to SPA use. Details are shown in **Table 3**.

**Table 3:** Detailed results on psychoactive substance use.

Variables		n	%
Age of first exposure to psychoactive substances (years)	Under 10	17	0.7
	10–12	110	4.5
	13–14	562	23.0
	15–17	1,051	42.9
	18–21	461	18.3
	22–26	128	5.2
	27–30	41	1.7
	31–40	33	1.3
	41–63	9	0.4
What substances have you used throughout your life (order from most frequently used to least frequently used)?	Marijuana (with THC or CBD)	2,396	97.9
	MDMA/ecstasy	1,858	75.9
	Amphetamine	1,497	61.2
	Mephedrone or other synthetic cathinones	1,463	58.7
	Cocaine	1,385	56.6
	Psilocybin mushrooms	1,380	56.4
	LSD or other lysergamides	1,167	47.7
	Benzodiazepines	706	28.9
	Methamphetamine	644	26.3
	Ketamine	597	24.4
	Synthetic cannabinoids (e.g., MDMB-CHMINACA, 5F-ADB)	494	20.2
	Other opioids	374	15.3
	DMT or plants containing DMT	345	14.1
	Alkyl nitrites (“poppers”)	269	11.0
	GHB/GBL	190	7.8
	*Amanita muscaria*	177	7.2
	Oxycodone	166	6.8
	Morphine	147	6.0
	Mescaline	76	3.1
	Benzofurans (e.g., 6-APB, 5-MAPB)	54	2.2
	Heroin	59	2.4
	Fentanyl	42	1.7
	Aminorexes	7	0.3
	Other than those mentioned in the survey	365	14.9
What substances have you consumed in the past 12 months (order from most frequently used to least frequently used)?	Marijuana (with THC or CBD)	2,080	85
	MDMA/ecstasy	1,029	42.1
	Mephedrone or other synthetic cathinones	892	36.5
	Psilocybin mushrooms	803	32.8
	Cocaine	735	30.0
	Amphetamine	633	25.9
	LSD or other lysergamides	505	20.6
	Ketamine	291	11.9
	Benzodiazepines	274	11.2
	Methamphetamine	239	9.8
	Other than those mentioned in the survey	153	6.3
	Other opioids	139	5.7
	DMT or plants containing DMT	138	5.6
	Synthetic cannabinoids (e.g., MDMB-CHMINACA, 5F-ADB)	114	4.7
	Alkyl nitrites (“poppers”)	68	2.8
	*Amanita muscaria*	64	2.6
	Oxycodone	59	2.4
	GHB/GBL	58	2.4
	Morphine	39	1.6
	Mescaline	20	0.8
	Benzofurans (e.g., 6-APB, 5-MAPB)	17	0.7
	Heroin	14	0.6
	Fentanyl	9	0.4
	Aminorexes	1	0.0
How often do you use psychoactive substances?	Once every few months or less frequently	872	35.6
	Once a month	275	11.2
	2–3 times a month	314	12.8
	4–5 times a month	157	6.4
	6–8 times a month	120	4.9
	9–10 times a month	75	3.1
	More than 10 times a month	634	25.9
How often do you take drugs other than alcohol?	Never	103	4.2
	Once a month or less often	1,042	42.6
	2–4 times a month	528	21.6
	2–3 times a week	277	11.3
	4 times a week or more often	497	20.3
Do you take more than one type of drug at a time?	Never	1,081	44.2
	Once a month or less often	1,030	42.1
	2–4 times a month	223	9.1
	2–3 times a week	61	2.5
	4 times a week or more often	52	2.1
With whom do you use drugs most often?	With friends	1,228	50.2
	By yourself	628	25.7
	With a partner/partner	525	21.5
	With family	18	0.7
	Other than the above	48	1.9
Where do you use psychoactive substances most often?	At home	1,280	52.3
	At someone’s home (including house parties)	527	21.5
	Outdoors (forests, meadows)	278	11.4
	In clubs	202	8.3
	At music festivals	137	5.6
	In bars	23	0.9
Where do you buy your psychoactive substances most often?	From friends	1,155	47.2
	From a dealer or from strangers at music festivals/clubs or bars	618	25.3
	In the darknet	130	5.3
	Other options not included in the survey	544	22.2
Do you test your psychoactive substances with colorimetric reagents or send them to a lab for testing?	No, never	2,045	83.6
	Sometimes	325	13.3
	Yes, always	77	3.1
How much do you spend on psychoactive substances in a month?	Up to 100 PLN	761	31.1
	100–200 PLN	367	15.0
	200–300 PLN	248	10.1
	300–400 PLN	233	9.5
	More than 500 PLN	416	17.0
	I don’t spend money. I get it from someone.	422	17.2
Do you measure the doses of your psychoactive substances?	Yes, always	651	26.6
	Sometimes	300	12.3
	Mostly by visual observation (“eyeballing,” by eye)	1,258	51.4
	Never	238	9.7
Do you use dietary supplements to support recovery after using psychoactive substances?	Yes, always	723	29.5
	Yes, sometimes	903	36.9
	No	821	33.6
Do you educate yourself about the safety and risks of psychoactive substances?	Yes	2,117	86.5
	No	330	13.5
Have you ever been in trouble with the law for using substances psychoactive substances?	Yes	410	16.8
	No	2,037	83.2
Have you ever neglected your daily responsibilities (work, study, family life) by taking psychoactive substances?	No, never	979	40.0
	Yes, once	243	9.9
	Yes, several times	1,012	41.4
	Yes, it happens to me often	213	8.7

The majority of respondents did not consider seeking professional help for psychoactive substance use (70.7%), while 221 respondents (9.0%) sought medical help soon after using psychoactive substances, most often after using mephedrone or other synthetic cathinones (32.6%). Some respondents(10.4%) are unable to specify after using what type of substance they sought medical help. Detailed results are shown in **Table 4**.

**Table 4:** Detailed results on professional assistance related to substance abuse.

Variables		n	%
Have you thought about seeking professional help for your psychoactive substance use?	Yes	718	29.3
	No	1,729	70.7
Have you ever sought medical help shortly after taking psychoactive substances (e.g., acute poisoning)?	Yes	221	9.0
	No	2,226	91.0
After what psychoactive substance did you seek medical help (n = 221)?	Mephedrone or other synthetic cathinones	72	32.6
	Amphetamine	57	25.8
	Marijuana	31	14
	Benzodiazepines	24	10.9
	MDMA/ecstasy	23	10.4
	I don’t know what substance I took	23	10.4
	Other opioids	18	8.1
	Cocaine	14	6.3
	LSD or other lysergamides	11	5.0
	Oxycodone	11	5.0
	Other	11	5.0
	Psilocybin mushrooms	9	4.1
	Fentanyl	7	3.2
	Morphine	6	2.7
	Heroin	6	2.7
	Synthetic cannabinoids (e.g., MDMB-CHMINACA, 5F-ADB)	6	2.7
	*Amanita muscaria*	2	0.9
	Alkyl nitrites (“poppers”)	1	0.5

Among the respondents, 1,055 people (43.1%) received psychiatric treatment, most often seeking help from a private practice (71.8%). Most respondents had been to a doctor for depressive disorders (50.0%). A total of 433 people (41.1%) in this group had attempted suicide at least once. In most cases, the first visit to the doctor was preceded by the use of psychoactive substances (70.5%). Most psychiatrists asked about the psychoactive substances used (79.3%). Only 40% would tell the doctor the truth about their psychoactive substance use regardless of the circumstances. Details are shown in **Table 5**.

**Table 5:** Detailed results on psychiatric treatment.

Variables		n	%
Have you received psychiatric treatment?	Yes	1,055	43.1
	No	1,392	56.9
For what reason? For various diagnoses, please mark the first one that was made (n = 1,055, order from most frequent answer to most rare)	Depressive disorders	528	50.0
	Anxiety disorders	156	14.8
	ADHD	105	9.9
	Addiction treatment	64	6.1
	Personality disorders	58	5.5
	Bipolar affective disorder	53	5.0
	Adaptive disorders	19	1.8
	Insomnia	15	1.4
	Schizophrenia	5	0.5
	Other	52	4.9
In what location did the treatment take place (n = 1,055)?	Private practice	758	71.8
	Mental health outpatient clinic	358	33.9
	Psychiatric ward closed	185	17.5
	Addiction treatment clinic	130	12.3
	Psychiatric day ward	55	5.2
	Other	35	3.3
Have there ever been any suicide attempts (n = 1,055)?	Yes, once	196	18.6
	Yes, several times	237	22.5
	No	622	58.9
Did psychiatric treatment precede the onset of psychoactive substance use (n = 1,055)?	Yes, I started using psychoactive substances after my first visit to a psychiatrist	311	29.5
	No, I went to a psychiatrist after I started using psychoactive substances	744	70.5
Did the psychiatrist ask about the psychoactive substances used during the interview collection (n = 1,055)?	Yes	837	79.3
	No	218	20.6
When going to a psychiatrist, would you tell the truth about the psychoactive substances used?	Yes, always	981	40.1
	Rather yes, if the doctor inspired my confidence	1,190	48.6
	No, I’m afraid of the legal consequences	61	2.5
	No, I’m afraid of being judged	75	3.1
	No, I don’t want to have anything “in the papers”	92	3.6
	No, for other reasons	48	2.0
Would you answer the same questions if you received a paper survey?	Yes	1,890	77.2
	No, filing online is more convenient	225	9.2
	No, the Internet provides a sense of anonymity	277	11.3
	No, for other reasons	55	2.2

## Discussion

The survey provides important data in the area of harm reduction. Compared to previous years, currently, more people declare problems with the law or neglect their daily duties, which may suggest an increase in the social harm of using psychoactive substances. Also, of concern is the increase in the percentage of people who mostly measure doses of their psychoactive substances “by eye” (40.9% in 2023 and 51.4% in 2025) and do not test them with colorimetric reagents or send them to a laboratory for testing. It is likely that this result is related to the increased popularity of stimulants and the increased percentage of people buying their psychoactive substances in conditions not conducive to accurate dosage measurement, i.e., from a dealer or from strangers at music festivals/clubs or bars. What is more, changing sources of substance acquisition—a decline in purchases from acquaintances (from 57.4% in 2023 to 47.2% in 2025) in favor of transactions with strangers—is reducing access to informal knowledge on harm reduction previously passed down orally in user communities. The rise in the popularity of buying stimulants from strangers may be influenced by the popularization of shopping via technologies that provide anonymity and encryption of Internet traffic (the so-called darknet), with the consequence that inexperienced individuals may increase their risk and expose themselves to dangerous situations more often. For this reason, activities seem particularly important at the grassroots: party working and street working, involving the presence of NGOs where drugs are consumed, that is, at parties and on the streets. It is also important to reduce the fear of testing psychoactive substances using home methods, such as colorimetric testing, by educating about the legality of such solutions. In addition, one can note the growing popularity of substance use in solitude (18.2% in 2021, 20.2% in 2023, and 25.7% in 2025). There is no clear interpretation of this trend. It is possible that this pattern of consumption is due to an increased post-pandemic tendency toward social isolation, especially among young adults. However, it cannot be ruled out that the type of substance consumed also has an impact. For example, a decline in interest in alcohol, which in Poland is one of the stimulants most strongly associated with social interaction, has been observed.

It is important to highlight that the minors also use illicit drugs. While the 13- to 17-year age subgroup comprises only 39 individuals, limiting its statistical power, we maintained its inclusion in the overall analysis to highlight the presence of minors in the dataset recognizing the relevance of their drug use patterns.

Polyuse of drugs presents a significant challenge in understanding substance use in society, as it blurs the distinctions between users of different substances. Individuals often consume multiple drugs simultaneously or sequentially making it difficult to categorize them based on a single substance. This overlap complicates both research and intervention efforts, as patterns of use and associated risks are not easily attributed to one specific drug.

Most respondents (70.7%) did not consider seeking professional help for psychoactive substance use, but 221 individuals (9.0%) reported seeking medical assistance shortly after use—most often following mephedrone or other synthetic cathinones (32.6%), a sharp rise compared to 2023 (19.4%) and 2021 (22.5%). Previously, amphetamine led in related medical interventions, but its numbers have remained stable (2025—25.8%; 2023—23.6%; 2021—22.5%), now surpassed by mephedrone. These trends align with data from the European Monitoring Center for Drugs and Drug Addiction showing an increase in synthetic cathinones as the most used substance (2021—44%; 2023—48%; 2025—58.7%) (13). The overall number of users requiring immediate medical help has also risen (2021—6.0%; 2023—6.4%; 2024—9.0%) likely due to the growing popularity of synthetic cathinones. Their unpredictable effects stem from limited knowledge of their metabolism and toxicity, frequent changes in composition, and identical appearance—making accurate identification and dosing difficult. For instance, a strong intranasal dose of mephedrone ranges from 80 to 125 mg, while for the nearly identical-looking pentedrone, it is only 10 to 20 mg (14, 15). Buying from unverified sources raises the risk of receiving the wrong substance increasing chances of overdose. In 2022, synthetic cathinones made up 87% of all new psychoactive substances seized in the EU—26.5 tons, up from 4.5 tons in 2021. The most common compounds were 3-CMC (63%), 3-MMC (9%), 2-MMC (5%), and N-ethylnorpentedrone (3%). Changes in alcohol consumption have been observed. In this year’s edition, 9.5 percentage points more respondents reported abstaining compared to those of 4 years ago. Among drinkers, the proportion consuming alcohol once a month or less increased (28.3% vs. 19.5% previously). Beer remains the most popular alcoholic beverage, though its use declined (from 56.3% in 2021 to 48.2% this year). More respondents also reported considering help for their alcohol use aligning with trends noted by the National Center for Addiction Prevention. One key factor affecting alcohol consumption is economic availability (16). This year’s sample included more people earning over 5,000 PLN net likely due to minimum wage increases and inflation. Since January 2022, Poland has raised excise taxes on alcohol, and in 2023, for the first time since 2009, fewer bottles of beer could be bought with the average salary. Nighttime alcohol sale restrictions and the growing popularity of non-alcoholic beers may also be contributing factors. According to the Association of Brewing Industry Employers—Polish Breweries, in the first half of 2024, alcoholic beer sales fell by nearly 1%, while non-alcoholic beer sales rose by over 16% (17). The change in the consumption profile is particularly evident in the group of young people living in large cities, and this is the group most strongly represented in our study (18). Particularly among young people, there is also an increase in awareness of the harmfulness of alcohol and trends such as “sober curious,” encouraging people to reduce alcohol consumption and experiment with a sober lifestyle (19). It is worth noting that alcohol is treated as a part of culture and national heritage in Poland, so these results are especially surprising. It is worth taking this trend into account when analyzing the data on the higher frequency of considering the use of help due to alcohol consumption than in previous years. It is possible that users are using alcohol in a more problematic manner, while on the other hand, public awareness of mental health and the specifics of addiction is increasing.

Loud media reports in June, July, and August 2024 increased opioid consumption, but the results of the survey do not confirm this information (20–23). The consumption of heroin, morphine, and fentanyl in Poland remains stable, which is described in **Table 6**. In previous editions of the survey, the authors did not ask about oxycodone.

**Table 6:** Percentage of users of each opioid according to the PolDrugs survey over the years.

Opioid	Year		
	2021	2023	2025
Heroin	1.1%	0.5%	0.6%
Morphine	3.4%	1.9%	0.4%
Fentanyl	0.6%	0.8%	0.4%
Oxycodone	No data available	No data available	2.4%

These trends are consistent with general patterns observed in the European Union (24). It is estimated that 0.3% of the EU’s adult population—approximately 860,000 people—were using opioids in 2022 compared to 950,000 in 2021. However, this apparent decline is likely not a reflection of a real decrease in opioid use across the EU. Rather, it is primarily due to a change in the statistical methodology used to estimate the number of heroin users in Italy, which likely has artificially lowered the overall figure. However, unlike most countries in the European Union, heroin is not the most commonly used illicit opioid in Poland, nor does the study show the European standard that heroin accounts for a significant portion of the health burden associated with drug consumption. Rather, synthetics are the main problem in Poland. Despite this, the opioid threat in Europe continues to evolve, which could have important implications for future drug policy strategies. Currently, synthetic opioids play a relatively minor role in the European drug market, but in some countries, they are already beginning to pose a serious challenge. Experts predict that their importance could increase, especially if the availability of heroin in Europe declines as a result of the Taliban’s ban on opium production in Afghanistan. The United Nations Office on Drugs and Crime (UNODC) estimated that poppy cultivation in Afghanistan declined by 95% following the ban in 2023. Given the potential consequences of such a market shift, the report’s authors recommend that Europe increase its preparedness for the challenges of harm reduction and control of new threats. However, an analysis of the results of PolDrugs 2025 does not indicate symptoms of this trend in Poland—at least at this stage.

Despite a growing number of medical studies on psychedelics, both in Poland and globally, our data show a noticeable decline in their reported use. Psilocybin mushroom consumption dropped from 42.0% in 2023 to 32.8% in 2025 (compared to 28.9% in 2021). LSD and other lysergamides followed a similar trend falling from 39.2% in 2021 to 20.6% in 2025. DMT use remained relatively stable, with a slight decline from 6.8% in 2021 to 5.6% in 2025. This may reflect shifting user preferences, possibly influenced by rising living costs, which reduce spending on more expensive or less euphoric substances. Another factor may be the fading media interest in psychedelics, despite increased scientific attention, contributing to a broader decline in public enthusiasm (25). We also noted a decline in the popularity of MDMA use. Use rates were 59.2% in 2021, 52.6% in 2023, and then dropped to 42.1% in 2025. One possible reason for this trend may be the FDA’s June 2024 decision to reject MDMA-assisted therapy for PTSD, which may have affected perceptions of the substance’s safety and efficacy (26). At the same time, the decline in MDMA’s popularity may be influenced by the observed increase in the popularity of mephedrone Due to the similar subjective effect among users of the substance, there may be some competition between MDMA and synthetic cathinones, especially in the context of club culture.

Unfortunately, although there is a decline in the number of people smoking classic cigarettes (from 37.4% in 2021 to 23.8% in 2025), the overall percentage of nicotine users remains stable (67.3% in 2025, 63% in 2023, and 66.8% in 2021). E-cigarettes (liquids) are a particularly popular form of taking it. While it is quite popular among users to believe that this is a healthier form of nicotine intake, this is not supported by scientific data. However, there is credible evidence that the aerosol from e-cigarettes contains harmful metals, the concentration of which may be higher than in traditional cigarettes (27). Some hope for shaping public health in this area is the fact that on 21 February 2025, the Polish parliament passed an amendment to the law banning the sale of heated tobacco products with a distinctive flavor. Work is also underway to ban the sale of nicotine pouches and all types of electronic cigarettes to minors, regardless of whether the product contains nicotine or not. There are also plans to extend the ban on the use of e-cigarettes containing nicotine in public places to those that do not.

In recent years, a novel trend has emerged where individuals who use recreational drugs are increasingly turning to dietary supplements in an attempt to mitigate adverse effects or enhance recovery. To our knowledge, this study is the first to directly ask drug users about this behavior marking a critical step in understanding its prevalence and implications. The growing visibility of this practice on social media platforms underscores the need for further research to evaluate both the safety and potential benefits of such supplementation strategies. Assessing the scientific validity and potential risks associated with this trend is essential to inform public health recommendations and harm reduction strategies.

Available data suggest changes in the structure of psychiatric care in Poland involving a reduction in inpatient hospitalization in favor of the development of community and day care. This is in line with the reform of psychiatry, conducted according to the model recommended by the World Health Organization (WHO), which aims to deinstitutionalize, destigmatize, and develop Mental Health Centers (MHCs) (28, 29). An analysis of National Health Fund reporting data from 2010 to 2014 indicates a steady increase in the number of services for mental disorders (30). Moreover, the increase in the number of patients treated for mood disorders, neurotic disorders, stress-related disorders, somatization disorders, and addiction disorders is particularly noticeable (30). According to an analysis conducted between 2017 and 2020, the percentage of patients receiving community care increased by 86% and 62% from outpatient care (31). The observed increase suggests that patients are more likely to use locally available services supporting earlier intervention and potentially reducing the need for hospitalization. The shift from inpatient to outpatient and community-based care enhances access to psychiatric services, strengthens doctor–patient relationships, and promotes a more personalized treatment approach. Survey results indicate growing awareness and acceptance of psychiatric help, with more individuals seeking support and reporting greater honesty with specialists—signs of increasing public trust in medical professionals. At the same time, there is a decline in hospitalizations in favor of day wards and addiction clinics reflecting the effectiveness of outpatient care. Additionally, physicians are showing more interest in patients’ psychoactive substance use indicating heightened clinical awareness. Notably, only 77% of respondents said they would answer the same questions on paper supporting the value of our online survey method. This is all the more interesting because, according to the Digital Poland survey, as many as half of Poles do not feel safe online, mainly due to concerns about data leakage and lack of trust in privacy protection (32).

### Limitations

The study is not free of limitations. It should be taken into account that due to the selection of respondents, our survey is based on volunteers with a non-probabilistic panel. We are dealing with the placement of the questionnaire online and the lack of control over who fills it out. However, due to the sensitive nature of the subject matter covered, freedom of expression is a key issue. The results come from people who use social media extensively, and it is certain that there is a group of recreational users of psychoactive substances who do not use Facebook or Instagram, probably people in older age groups. For this reason, there are limitations to the representativeness of our sample. Nevertheless, the fact that the results can be compared with those of 2 and 4 years ago, and the fact that social media is a very large part of daily life, encourages us to publish the above results.

## Conclusions

The findings of our study highlight several emerging trends in drug use and mental health in society. Notably, the use of stimulants—particularly mephedrone and its derivatives—is increasing, alongside a rising number of individuals seeking medical help following their use. This pattern warrants careful monitoring and further investigation. Additionally, we observed a gradual, year-on-year increase in the proportion of individuals who use psychoactive substances alone, with this group now accounting for more than one quarter of all respondents. Interestingly, the number of psychedelic users appears to be declining, which may be linked to a reduction in mainstream media interest and coverage. Finally, the results support the observation that acceptance of psychiatric support is growing within Polish society reflecting a positive shift in attitudes toward mental healthcare.

## Data Availability

The raw data supporting the conclusions of this article will be made available by the authors, without undue reservation.
